# A Complex Case of Triumphantly Treated ​​​​​​Anti-N-Methyl-D-Aspartate (NMDA) Receptor Encephalitis

**DOI:** 10.7759/cureus.78341

**Published:** 2025-02-01

**Authors:** Amer Abu-Shanab, Raymart Macasaet, Ahmed B Mohd, David Aquino, Anoohya Vangala, FNU Payal, Doantrang Du

**Affiliations:** 1 Department of Internal Medicine, Monmouth Medical Center, Long Branch, USA; 2 Faculty of Medicine, Hashemite University, Zarqa, JOR

**Keywords:** anti nmda receptor encephalitis, encephalitis, intravenous immunoglobulin (ivig), neurology and psychiatric disorders, nmda receptor, teratoma

## Abstract

Anti-N-methyl-D-aspartate (NMDA) receptor encephalitis is a rare autoimmune disorder characterized by neuropsychiatric symptoms and the presence of immunoglobulin G (IgG) antibodies against the N-methyl-D-aspartate receptor 1 (NR1) subunit of NMDA receptors. It is often triggered by ovarian teratomas, especially in young women, and presents with a combination of psychiatric and neurological symptoms. Here, we present a case of a 25-year-old woman with a history of schizoaffective disorder and post-partum depression who presented with two months of bizarre behavior, agitation, and decreased sleep. Initially admitted for suspected psychosis, her condition worsened with episodes of loss of consciousness and failure to respond to psychiatric medications. After extensive investigations, including computed tomography (CT), magnetic resonance imaging (MRI), and electroencephalogram (EEG), NMDA receptor encephalitis was suspected and confirmed by positive autoantibodies. Imaging revealed bilateral ovarian teratomas, and she underwent a cystectomy. The patient was treated with intravenous immunoglobulin (IVIG), which led to complications, including desaturation and the need for intubation. Initially, five IVIG sessions were given. Based on their experience, and due to sub-optimal recovery, a shared decision was made between the neurology team and the intensive care unit (ICU) team to administer two additional IVIG sessions. The single dose of each IVIG session was 35 g (0.4 g/kg). After seven sessions of IVIG, she showed gradual improvement. This case underscores the importance of considering NMDA receptor encephalitis in patients with unexplained neuropsychiatric symptoms and highlights the critical roles of autoantibody testing, tumor resection, and immunotherapy in the management of this condition.

## Introduction

Anti-N-methyl-D-aspartate (NMDA) receptor encephalitis is a rare autoimmune disorder characterized by neuropsychiatric symptoms and the presence of immunoglobulin G (IgG) antibodies against the N-methyl-D-aspartate receptor 1 (NR1) subunit of NMDA receptors in the central nervous system. While the exact cause is unknown, antibody production is sometimes triggered by ovarian teratomas or viral infections, such as herpes simplex virus encephalitis. The condition is associated with neurological symptoms, including seizures, dyskinesias, autonomic instability, and psychiatric symptoms that are the reason for presentation in most cases [1,2]. Most of the existing literature on treatment primarily addresses tumor removal and immunotherapy aimed at modulating the immune response. However, there is a lack of detailed guidance on managing the psychiatric symptoms in these patients, who are frequently in critical condition and present with complex clinical challenges [1]. In this case report, we present a complicated case of anti-NMDA receptor encephalitis that was treated successfully.

## Case presentation

A 25-year-old woman with a past medical history of schizoaffective disorder, post-partum depression, and obesity presented with a two-month history of bizarre behavior. She exhibited increased agitation, aggression, and irritability, with a marked reduction in sleep duration. She was screaming in the house for no reason, waking up in the middle of the night and crying, and mistaking her family members’ names. Initially, the patient was admitted to the psychiatric unit with suspected psychotic decompensation. However, during her stay, she experienced episodes of loss of consciousness, prompting her to transfer to the medical floor for further evaluation. A workup, including head computed tomography (CT), magnetic resonance imaging (MRI), lumbar puncture (LP), and electroencephalogram (EEG), was unremarkable. Given the patient's age, atypical presentation, and lack of improvement with psychiatric interventions, autoimmune encephalitis was suspected, specifically anti-NMDA receptor encephalitis.

**Figure 1:**
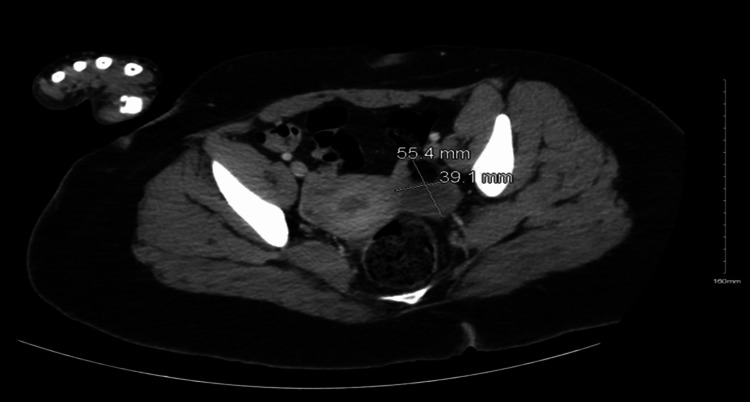
CT scan of the pelvis demonstrating a 55.4 mm by 39.1 mm left ovarian teratoma.

**Figure 2:**
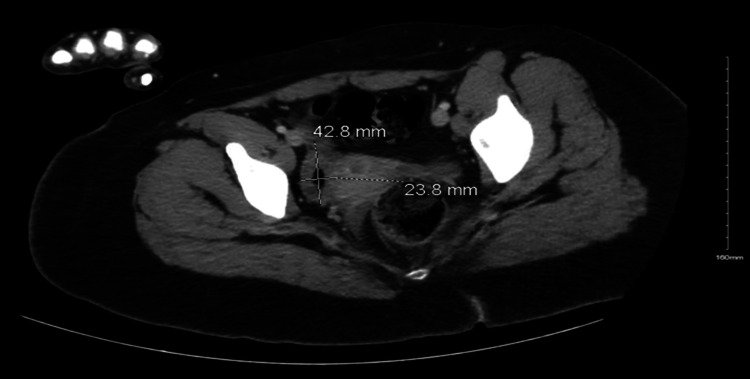
CT scan of the pelvis demonstrating a 42.8 mm by 23.8 mm right ovarian teratoma.

A CT scan of the abdomen and pelvis revealed bilateral teratomas, and subsequent testing confirmed the presence of NMDA receptor autoantibodies at a titer of 1:32. Figures 1, 2 demonstrate the teratomas seen on the CT scan. Following the diagnosis, the patient underwent cystectomy for the removal of the bilateral teratomas and was initiated on intravenous immunoglobulin (IVIG) therapy.

After the first session of the IVIG treatment, the patient experienced an aspiration event, leading to severe hypoxic respiratory failure, which required intubation and admission to the intensive care unit (ICU) for respiratory support. During her ICU stay, she received a total of five IVIG courses alongside broad-spectrum antibiotics, including vancomycin and piperacillin-tazobactam, due to concerns for potential meningitis. The patient showed significant, yet incomplete resolution of her encephalitis symptoms and she continued to require respiratory support. Based on their experience, a shared decision between the neurology team and the ICU team was made to administer two additional IVIG sessions. The single dose of each IVIG session was 35 g (0.4 g/kg). Following the seventh IVIG treatment, the patient’s respiratory status improved, and she began to regain consciousness.

In the ICU, the patient also experienced multiple episodes of prolonged cardiac pauses, ranging from three to eight seconds. The cardiology team recommended the implantation of a pacemaker, but this was deferred due to ongoing septic concerns. Once the patient’s septic condition improved, it was planned to schedule the pacemaker implantation during outpatient follow-up. She was successfully extubated and transferred out of the ICU for continued management.

On the floors, the patient started physical therapy and then was transferred to an acute rehabilitation facility. She demonstrated gradual improvement and was able to regain the ability to walk. Her rehabilitation plan continued as she progressed, and she was scheduled for follow-up care in the outpatient clinic.

## Discussion

This case highlights the diagnostic and therapeutic challenges associated with NMDA receptor encephalitis, a condition that can present with predominantly neuropsychiatric symptoms and pose significant management hurdles. The patient, a 25-year-old woman with a complex medical history, exhibited a constellation of symptoms that included agitation, aggression, and altered consciousness, which initially led to admission for suspected psychotic decompensation, thus psychiatric admission.

The patient's presentation with bizarre behavior and loss of consciousness, coupled with inconclusive results from standard neuroimaging and EEG, with no improvement after trying different anti-psychotic medications, necessitated consideration of less common diagnoses. In this case, the positive NMDA receptor autoantibody titer (1:32) was instrumental in guiding the diagnosis, reflecting the critical role of autoantibody serology in identifying autoimmune encephalitis, mainly when initial investigations are unremarkable. The presence of psychiatric medical conditions in the patient complicates the diagnosis even more. Our patient was initially admitted to the psychiatric unit for three weeks, given her history of schizoaffective disorder and post-partum depression, before being transferred to the medical floors for further workup.

The identification of bilateral teratomas in this patient underscores the well-established association between NMDA receptor encephalitis and ovarian teratomas [3]. Teratomas are known to be a common tumor type associated with NMDA receptor encephalitis in young women, and their resection is a crucial component of the treatment strategy [3]. The patient's response to cystectomy highlights the importance of tumor removal in improving outcomes for patients with paraneoplastic syndromes. Ovarian teratomas can be treated using various methods, such as oophorectomy or cystectomy. The choice of method should consider factors like the patient's age, family planning, and the disease's severity. In this case, the patient was young, the cysts were visible, and we did not have consent from the family for oophorectomy. Consequently, we opted for an initial cystectomy. Since the patient showed improvement, oophorectomy was not required.

Cardiac complications, including cardiac pauses, are notable in NMDA receptor encephalitis, reflecting the disorder's impact on autonomic regulation and cardiac function. Patients with NMDA receptor encephalitis may experience arrhythmias, such as bradycardia and tachycardia, due to dysregulation of the autonomic nervous system [1,3]. Cardiac pauses, or transient periods of asystole, can occur, potentially leading to symptoms such as syncope or dizziness. These complications are linked to the disease's broader systemic and inflammatory effects, which can disrupt normal cardiac rhythm and autonomic control [1]. This patient had frequent episodes of cardiac pauses, the longest was eight seconds, for which we planned on placing a pacemaker. Given the patient’s positive blood culture and the improvement in the patient’s symptoms, the procedure was deferred to outpatient.

The initiation of IVIG therapy, a cornerstone of treatment for NMDA receptor encephalitis, was complicated by severe adverse effects, including desaturation and the need for intubation. Despite that, we are not sure whether the IVIG was the direct cause of the respiratory failure or it was part of the disease course; this adverse reaction emphasizes the need for close monitoring during IVIG administration and the full course of the disease, as severe complications, although rare, can occur and require prompt intervention [2]. The use of IVIG, despite these complications, ultimately contributed to the patient's gradual improvement, reflecting its efficacy in managing NMDA receptor encephalitis [4]. We did five sessions initially; the patient improved significantly but not fully. Thus, we decided on two more sessions.

The gradual recovery following a full course of IVIG and the eventual extubation are encouraging and align with literature describing the progressive improvement seen in patients with NMDA receptor encephalitis after appropriate treatment [4]. This case reinforces the importance of a multidisciplinary approach in managing complex cases involving autoimmune encephalitis, integrating neurology, psychiatry, cardiology, surgery, and critical care to optimize patient outcomes.

## Conclusions

This case underscores the importance of considering anti-NMDA receptor encephalitis in patients with acute neuropsychiatric symptoms, particularly when traditional diagnostic methods are inconclusive. Young female patients are more prone to this condition. The association with teratomas, cardiac pauses, and the complex management of autoimmune therapy and critical care highlight key aspects of this challenging condition. IVIG was helpful in this case and extending the course from five to seven days showed a better outcome for our patient. Timely diagnosis and comprehensive treatment, including tumor resection and immunotherapy, are crucial for improving patient outcomes.
